# Supplementary material for: Lactation and menstruation shift the vaginal microbiota in captive rhesus monkeys to be more similar to the male urethral microbiota
Source: Sci Rep. 2019 Nov 22;9:17399. doi: 10.1038/s41598-019-53976-8 (PMC6874612; doi:10.1038/s41598-019-53976-8)
Supplement: Supplementary file 1 — Supplementary Material [file 41598_2019_53976_MOESM1_ESM.pdf]

**Supplementary Material**

# Lactation and menstruation shift the vaginal microbiota in captive rhesus monkeys to be more similar to the male urethral microbiota

L. K. Hallmaier-Wacker[1,2], S. Lüert[1,2], C. Roos[2,3], S. Knauf[1,4*]

(1) Work Group Neglected Tropical Diseases, Infection Biology Unit, Deutsches Primatenzentrum GmbH, Leibniz Institute for Primate Research, Goettingen, Germany.

(2) Primate Genetics Laboratory, Deutsches Primatenzentrum GmbH, Leibniz Institute for Primate Research, Goettingen, Germany.

(3) Gene Bank of Primates, Deutsches Primatenzentrum GmbH, Leibniz Institute for Primate Research, Goettingen, Germany.

(4) Division of Microbiology and Animal Hygiene, Georg-August-University, Goettingen, Germany.

*Address correspondence to Sascha Knauf, DVM, PhD, sknauf@dpz.eu, Tel. +49 551 3851 259

***Supplementary Figures:***

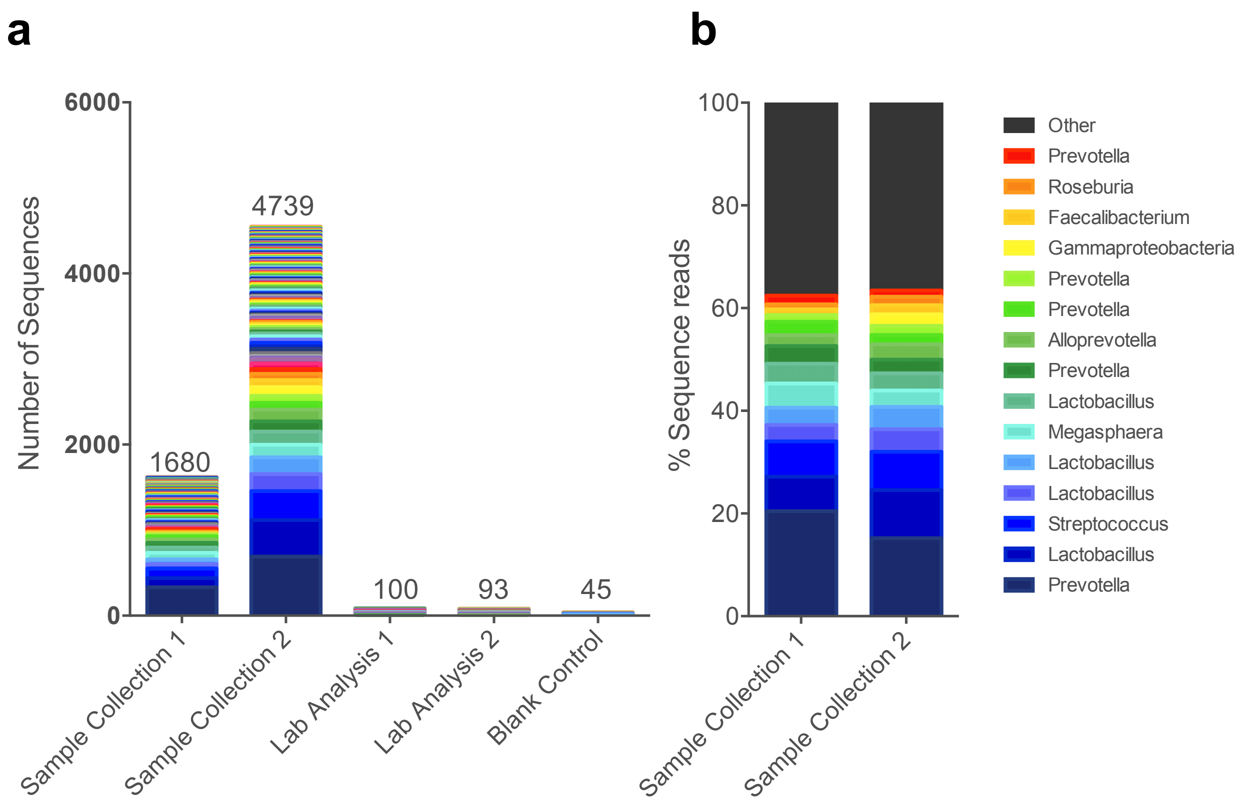


**Fig. S1: Bacterial profile of control samples included in this study.** (**a**) A total of 6,657 sequences corresponded to the control samples included during sample collection, lab analysis and 16S rRNA amplification (blank control). (**b**) Taxa plots showing the relative abundance of the 15 most abundant OTUs in percentage of reads for control samples taken during sample collection at two different breeding groups (sample collection 1: group A and sample collection 2: group E).

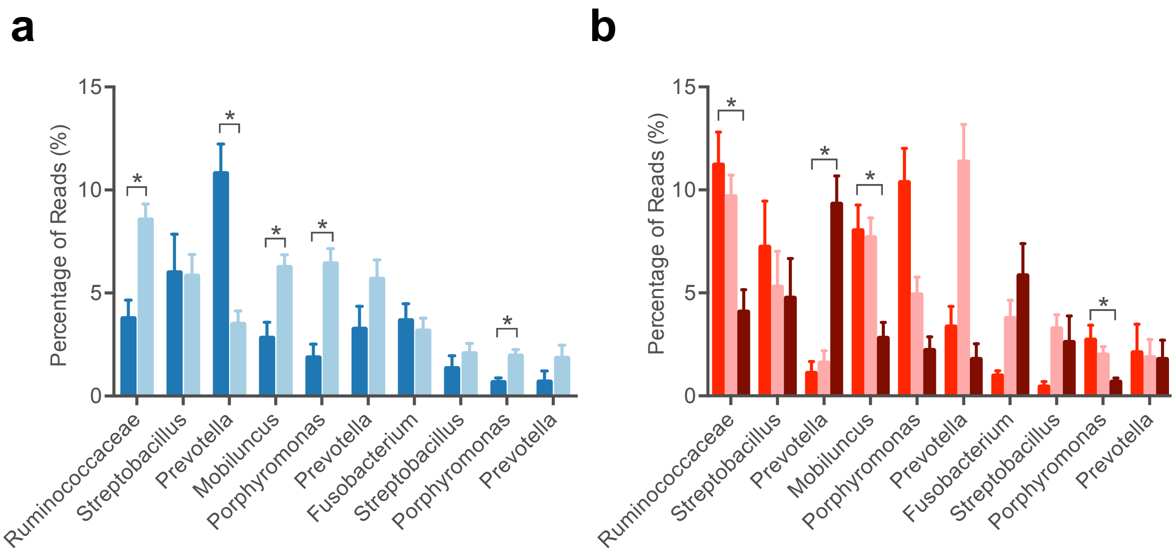


**Fig. S2: Individual bacterial OTUs are significantly different in the vaginal microbiota of lactating and menstruating female monkeys.** Percentage of sequence reads (mean ± SD) for the ten most abundant OTUs in the vaginal microbiota sorted by (**a**) lactation status (lactating (dark blue) and non-lactating (light blue) and (**b**) sexual cycle phases (P1: ovulatory phase (red), P2: intermediate stage (pink), P3: menstruation-like (dark red)). Differences in the ten most abundant OTUs were assessed using the metastats command in mothur (*$p \leq 0.001$; For (**b**) significance is shown only if P3 was significantly different for both P1 and P2).

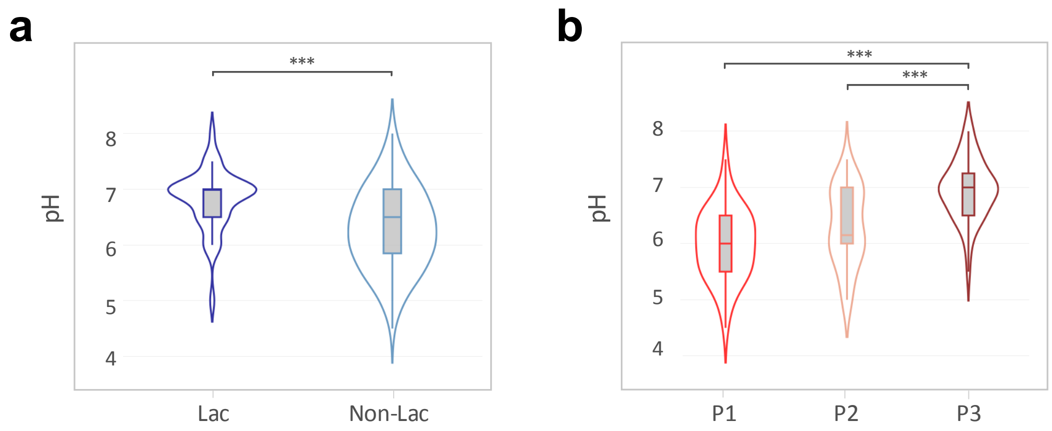


**Fig. S3: Vaginal pH was significantly different in lactating and menstruating animals.** Violin plots of the vaginal pH grouped by (**a**) lactation status (Mann-Witney t-test ***p≤0.0001) and (**b**) sexual cycle phases (P1: ovulatory phase, P2: intermediate stage, P3: menstruation-like; Kruskal-Wallis test ***p≤0.0001).

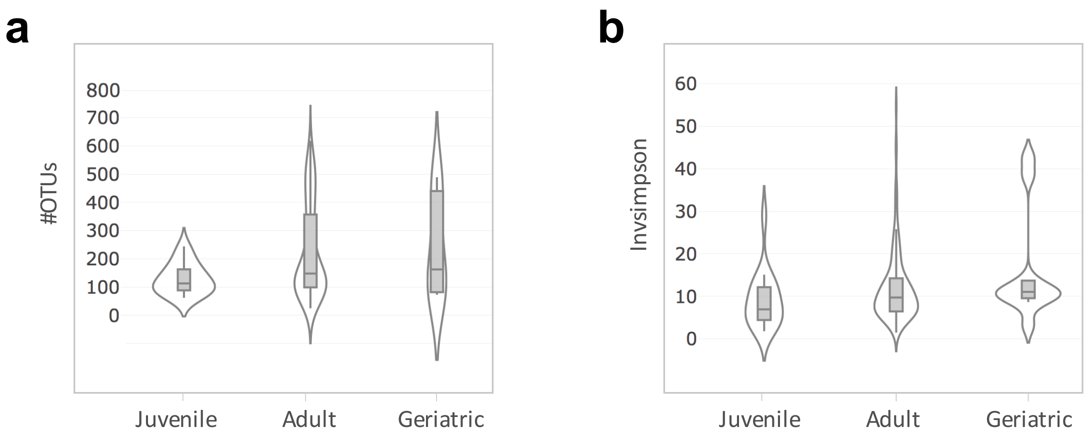


**Fig. S4: Alpha diversity measurements for vaginal microbiota of rhesus monkeys grouped by age composition.** Violin plots of the (**a**) observed OTUs and (**b**) InvSimpson index (Kruskal-Wallis test). See Table 1 for sample size composition and age classification.

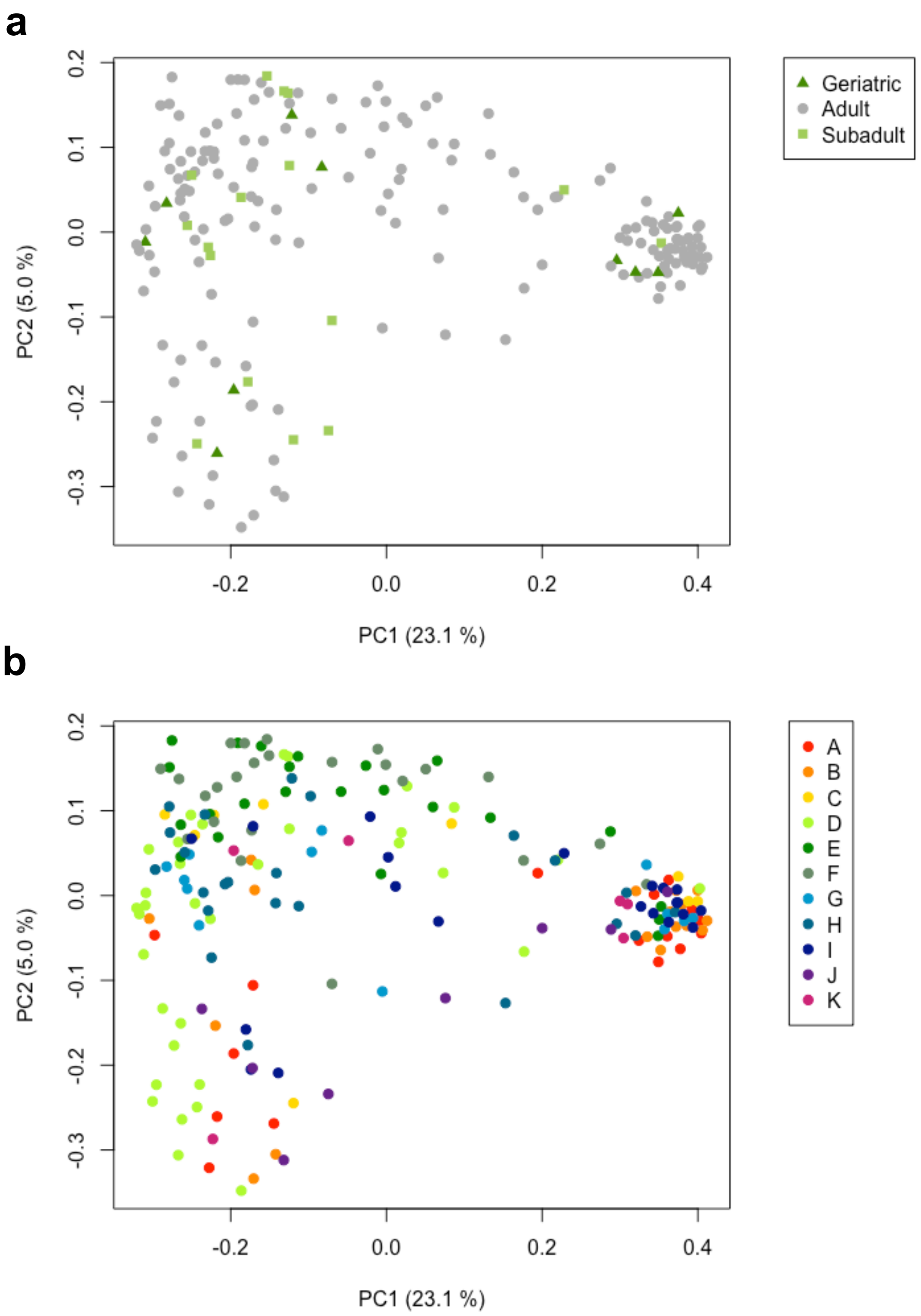


**Fig. S5: Principal coordinates analysis of vaginal samples separated by (a) age comparison and (b) group association.** Distances between samples were calculated using the unweighted UniFrac metrics. Figure legend shows age groups and group association (groups: A-K). See Table 1 for sample size composition and age classification.

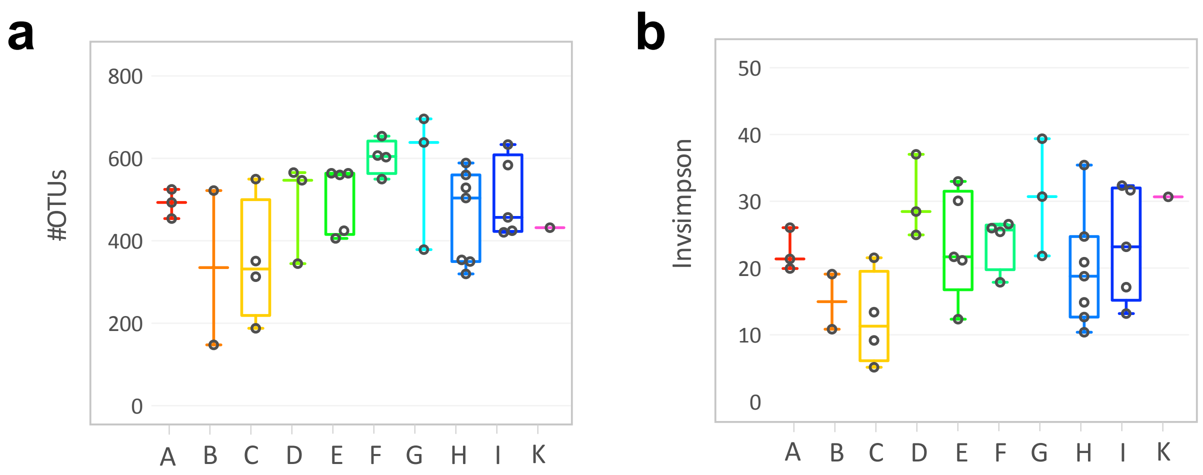


**Fig. S6: Alpha diversity measurements for urethral microbiota of male rhesus monkeys grouped by group composition.** Boxplots (median ± range) of the (**a**) observed OTUs and (**b**) InvSimpson index clustered.

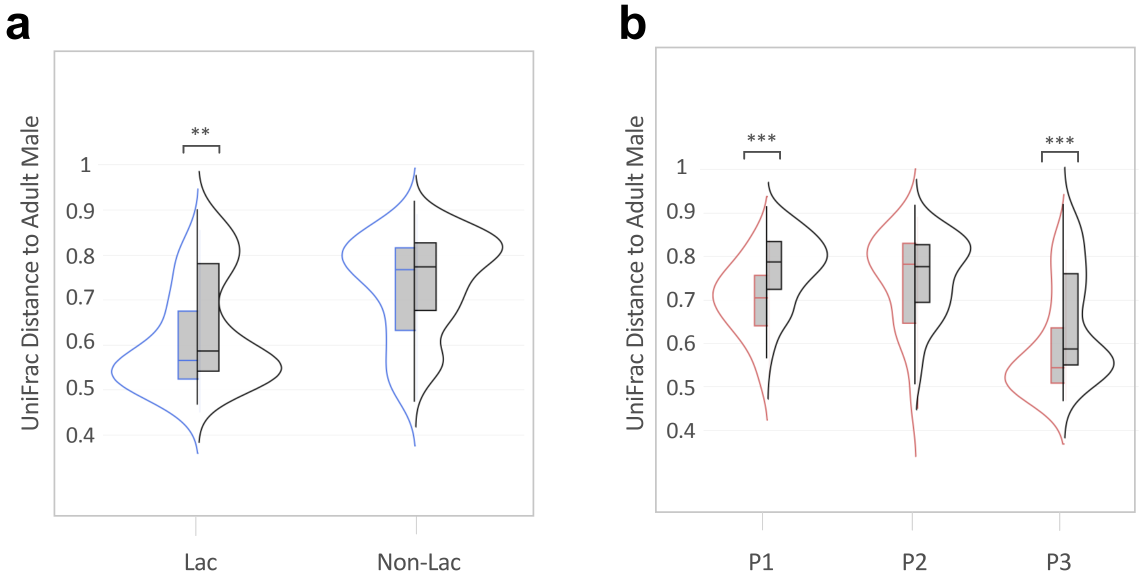


**Fig. S7: Cage-mate comparison between adult male and female genital microbiota subdivided by lactation status and sexual cycle phase.** Paired violin representations showing unweight UniFrac Distance of the adult vaginal microbiota to the urethral microbiota of the adult males. Data is sorted by (**a**) lactating status and (**b**) sexual cycle phase (P1: ovulatory phase, P2: intermediate stage, P3: menstruation-like). The colored (blue/red) violin plots correspond to the unweight UniFrac distance between the adult male and females in the same cage, while black plots correspond to the unweighted UniFrac distance between the alpha male and females from other breeding groups (no sexual contact possible) (Mann-Witney t-test ***p≤0.0001).

***Supplementary Tables:***

**Table S1: Maturating value and phase classification for exfoliative vaginal cytology used in this study**

| Phase Assignment | Maturation value | Sexual cycle phases | Cell composition | % Lac (n= 44) | % Non-Lac (n=104) |
|---|---|---|---|---|---|
| Phase 1 | 95-100 | Ovulatory | Dominated by superficial cells | 15.9% | 35.6% |
| Phase 2 | 45-95 | Intermediate | Intermediate, parabasal & basal cells | 31.8% | 41.3% |
| Phase 3 | >45 | Menstruation-like* | Diverse including cellular debris & blood cells (erythrocytes and leucocytes) | 52.3% | 23.1% |

*Menstruation-like: this phase represents both menstruation and postpartum amenorrhea

**Table S2: Reagents and kits used in this study with lot numbers and suppliers**

| Reagent | Supplier | Lot Number |
|---|---|---|
| FLOQSwabs, regular | Copan Improve Diagnostics | F5RM00 |
| FLOQ swabs, mini-tip | Copan Improve Diagnostics | 8H0D00 |
| Microtube SafeSeal, 1.5 ml | Sarstedt AG & Co. | 7080311 |
| DNA LoBind Tubes, 1.5 ml | Eppendorf AG | G171343G |
| DNA LoBind Tubes, 2.0 ml | Eppendorf AG | D157963O |
| The QIAamp Mini Kit | Qiagen GmbH | 157033520 |
| Microbial DNA-Free water | Qiagen GmbH | JE01 |
| Phusion HS II HF DNA Polymerase | Thermo Fisher Scientific | 00607540 |
| AMPure XP beads | Beckman Coulter | 16909300 |
